# Bullous Henoch-Schönlein Purpura and Associated Nephritis: A Pathological Case Report

**DOI:** 10.7759/cureus.35051

**Published:** 2023-02-16

**Authors:** Hristo Popov, Tatiana Koleva, George S Stoyanov

**Affiliations:** 1 General and Clinical Pathology, Forensic Medicine and Deontology, Medical University of Varna, Varna, BGR; 2 Nephrology, St. Marina University Hospital, Varna, BGR; 3 General and Clinical Pathology, St. Marina University Hospital, Varna, BGR

**Keywords:** immunofluorescence, glomerulonephritis, nephropathology, bullous dermatosis, henoch-schönlein purpura

## Abstract

Henoch-Schönlein purpura (HSP) is the most common vasculitis in childhood, presenting with purpura, predominantly of the lower extremities and occasionally with renal involvement as well. Although associated with childhood, HSP, although rarely, can also develop in adults as well. Here we present a patient in his sixties, presenting with a myriad of rash units on his lower extremities, including bullous ones, and a constellation of chronic kidney failure. Skin and renal biopsy specimens revealed morphological changes and immune depositions representative of HSP. Despite treatment, the patient's kidney failure slowly progressed, and he expired eight months after his presentation due to associated complications. Although rare, the bullous form of HSP can be viewed as a more aggressive form of the disease, as seen by the presentation constellation and rapid progression in our case.

## Introduction

Henoch-Schönlein purpura (HSP) or immunoglobulin A (IgA) vasculitis is the most common vasculitis disorder in childhood, with a yearly incidence of around 20 cases per 100,000 capita in the pediatric population aged 17 and younger, but it also occurs in adults [1]. Although the disease is usually a self-limiting one, and hence with a favorable prognosis, it can, in the context of renal failure, lead to permanent organ damage in the form of severe nephritis [2-5]. Hence HSP nephritis (HSPNis a major prognostic factor for patient outcome as it develops in around 20-60% of IgA vasculitis (IGAV)/HSP pediatric patients [2-7].

As the diagnosis of HSPN requires a kidney biopsy, the spectrum of histological changes observed is key in establishing the patient's prognosis and determining the treatment of choice. The majority of children with IGAV/HSP (97%) develop kidney-related symptoms within the first six months of disease onset, but occasionally HSPN may develop significantly later [5, 8-10]. The spectrum of kidney-related symptoms ranges from urinary abnormalities such as hematuria and/or proteinuria through nephritic and nephrotic syndrome to chronic renal failure.

The most typical presentation of renal disease in HSP is usually mild and is manifested only by pathological findings in the urine, with most patients, about 50%, developing only concurrent hematuria and proteinuria [8, 11]. On the other hand, more severe changes associated with the development of nephritic or nephrotic syndrome occur in about 20% of patients [8,11].

A rare clinical form of manifestation of HSP is the initial onset of hemorrhagic vesicles and bullae, leading to skin necrosis, a bullous form of HSP [12]. Furthermore, initial presentation with both cutaneous involvement and severe clinical and morphological changes in the renal parenchyma is also a rare feature of the condition with a typical dismal prognosis.

## Case presentation

A 67-year-old male presented with complaints of vertigo accompanied by shortness of breath, general weakness, abdominal pain, and severe lower extremity pain (arthralgia) with swelling. Clinical examination revealed multiple raised, confluent rash units - macules, papules, and vesicles on lower limbs. Some of the vesicles had ruptured and led to the formation of ulcers with concomitant excoriations, with further similar evolution of the vesicular rash.

Laboratory tests revealed a constellation of chronic kidney failure: 1.2 g of protein in the urine, gross hematuria, creatinine of 589 mcmol/l, and urea of 18.5 mmol/l. Blood cultures came back negative for infectious agents and serum complement levels and circulating antibodies were within normal range or negative.

As HSP was suspected, the patient was referred for a biopsy of a fresh skin lesion and a kidney biopsy. Previous medical history included hypertensive disease for the past 17 calendar years, under adequate medication control, but was otherwise uneventful, with the patient denying any additional medicational intake.

**Figure 1:**
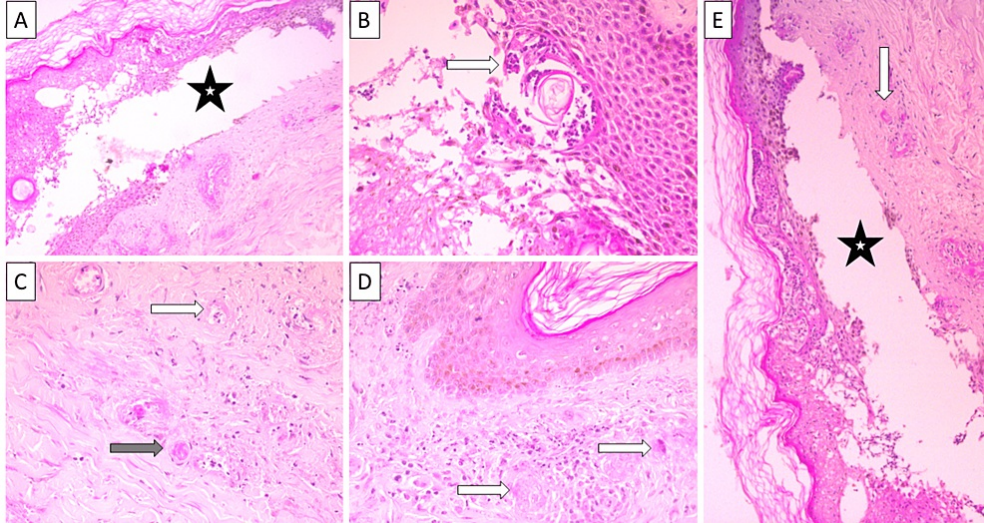
Histopathology of the skin biopsy A: bullous changes (star), H&E stain, original magnification 100x; B: eosinophilic infiltration (arrow), H&E stain, original magnification 400x; C: fibrinoid necrosis (arrow) and microthrombi (shaded arrow), H&E stain, original magnification 400x; D: fibrinoid necrosis and inflammatory infiltration, H&E stain, original magnification 400x; E: bullous change (star) and fibrinoid necrosis (arrow), H&E stain, original magnification 100x H&E: hematoxylin and eosin

**Figure 2:**
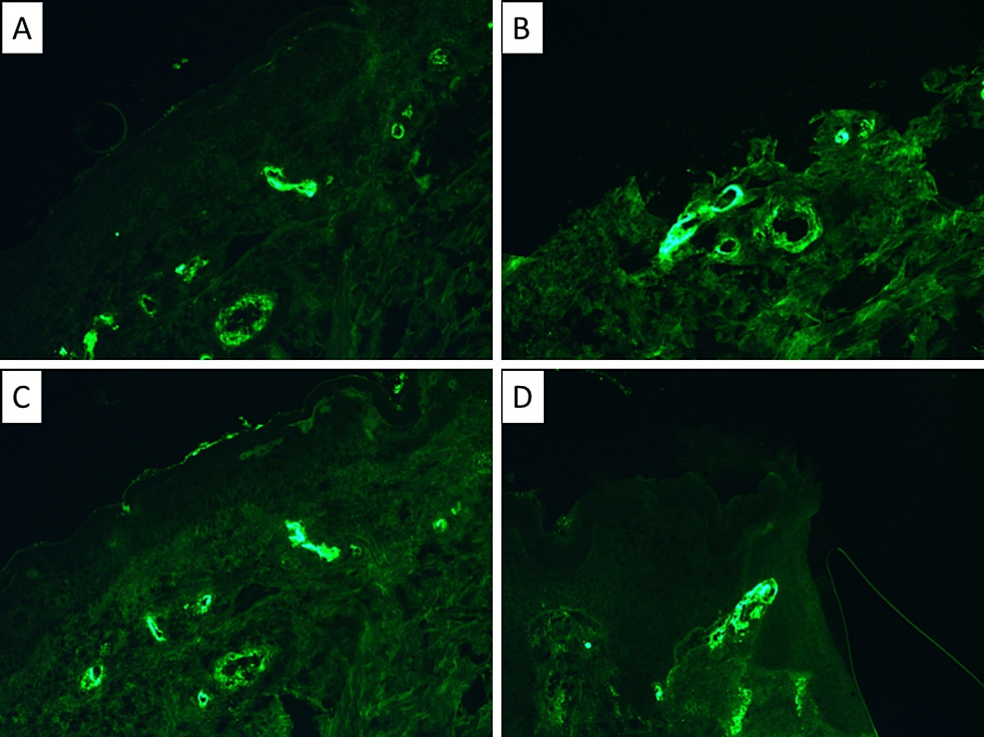
Immunofluorescent finding of the skin biopsy A: Immunoglobulin M, original magnification 400x; B: complement fraction 3, original magnification 400x; C: Immunoglobulin A, original magnification 400x; D: complement fraction 3, original magnification 400x

Light microscopy of the skin biopsy showed a subepidermal bulla with neutrophilic and eosinophilic leukocytes in the bullous space, keratinocyte spongiosis in the superficial dermis, the presence of blood vessels with fibrinoid necrosis, microthrombi, perivascular infiltrates of neutrophilic leukocytes, single lymphocytes, plasma cells and histiocytes (Figure 1). Immunofluorescence of the same skin sample showed abundant vascular wall deposits of IgA, immunoglobulin M (IgM), and complement fraction 3 (C3) (Figure 2).

**Figure 3:**
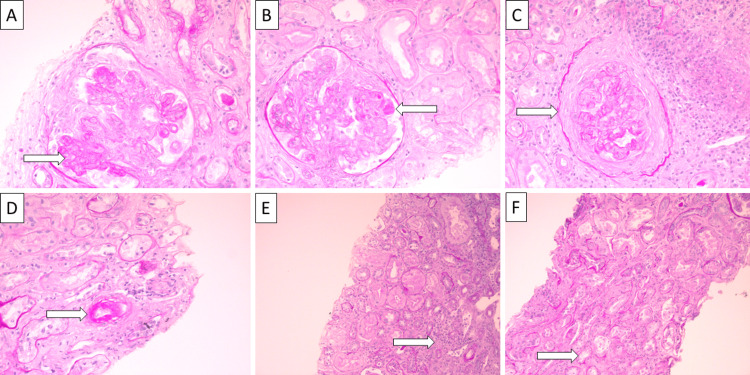
Light microscopy of the kidney biopsy A: glomerular collapse (arrow), PAS stain, original magnification 400x; B: microthrombi (arrow), PAS stain, original magnification 400x; C: glomerular crescents (arrow), PAS stain, original magnification 400x; D: fibrinoid necrosis (arrow), original magnification 400x; E: interstitial fibrosis and inflammation (arrow), PAS stain, original magnification 100x; F: tubular atrophy (arrow), PAS stain, original magnification 400x. PAS: Periodic acid–Schiff

**Figure 4:**
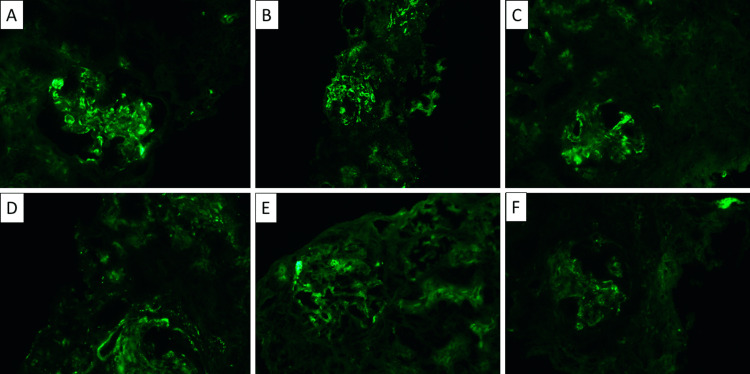
Immunofluorescent finding of the renal biopsy A: Immunoglobulin A, original magnification 400x; B: complement fraction 3, original magnification 400x; C: lambda chains, original magnification 400x; kappa chains, original magnification 400x; D: complement fraction 3 in blood vessels, original magnification 400x; E: lambda chains, original magnification 400x; F: immunoglobulin M, original magnification 400x

The renal biopsy specimen showed extra capillary proliferation forming cellular crescents, the collapse of capillary loops, fibrinoid necrosis and adhesions to the crescents, cortical fibrosis and tubular atrophy on light microscopy, and mesangial deposits of IgA, IgM and C3, the same as in the skin lesion, on immunofluorescence (Figures 3-4).

Despite systemic therapy with corticosteroids and cyclophosphamide, the patient had to undergo chronic hemodialysis six months after the presentation and expired two months after that due to chronic kidney disease-associated complications.

## Discussion

HSP usually affects the lower limbs and presents clinically with a petechial rash (purpura). The differential diagnosis in these cases typically includes congenital or acquired forms of bleeding disorders and other types of vasculitis, with biopsy conformation of the spectrum of changes being key. However, atypical forms of HSP can initially present with hemorrhagic vesicles and bullae, progressing to skin necrosis with ulceration, as seen in our case. These lead to significant diagnostic dilemmas and diagnosis and treatment initiation delay, which can further impact the patient's prognosis [12].

The differential diagnosis of bullous skin lesions typically includes bullous dermatoses (autoimmune diseases) such as pemphigoid and bullous pemphigus (Vulgaris), certain infections such as herpes simplex virus, genetic disorders such as epidermolysis bullosa and dermatitis herpitiformis, as well as vesicles and bullae caused by congestion and stasis, pressure or trauma [13]. 

While immune depositions in leukocytoclastic vasculitis are regarded as pathognomonic features on biopsy, IgA cannot always be detected in all cases. This is primarily due to biopsy timing and the stage of evolution of the biopsied lesion, as IgA deposition is more challenging to detect in older and treated lesions. This suggests that the biopsy should be taken from the border of a fresh and non-necrotized lesion, where the proteolytic degradation of IgA is less advanced. Our patient's skin biopsy was performed ten days after the appearance of the bullous lesions that were treated daily with antiseptics, but despite this, the deposition of IgA, IgM, and C3 in vessel walls was well demonstrated.

The long-term prognosis in HSP is mainly determined by the degree and severity of renal involvement [4]. As seen in our case, the patient had significant kidney changes associated with the disease on presentation, mesangial deposition for IgA, IgM, and C3, as well as crescents, microthrombi, and fibrinoid necrosis of blood vessel walls, and despite the initiation of treatment, the chronic kidney disease progressed rapidly. 

Systemic use of corticosteroids as treatment in HSP appears to have no effect on the progression of kidney-related changes while relieving abdominal pain and arthralgia and reducing the prevalence of purpura [14-17]. Therefore the evaluation of kidney function and morphology especially can lead to early detection of these changes, treatment optimization, and improve the patient prognosis significantly [14-17].

The presented form of bullous HSP can be interpreted both as a rare manifestation of the disease and, as seen in our case, as a more severe disease form or even a distinct disease entity of an HSP variant [18].

## Conclusions

Unlike conventional purpura, bullous lesions rarely develop and are a presenting symptom of HSP. Furthermore, as our patient was an adult, another atypical factor in the presentation of the disease, this made the clinical differential diagnosis a comprehensive one. Cutaneous lesion and renal biopsy, based on the clinical and laboratory constellation of the case presented, however, proved the diagnosis to indeed be HSP. Sadly, despite the initiation of treatment, the condition progressed and the patient eventually expired from kidney-associated complications.

Bullous lesions in this aspect, as a rarely depicted component of HSP, may indicate a specific variety of the condition, more challenging to suspect clinically and, as seen in our patient, with a higher risk of progression and development of complications.
